# Prevalence of common hereditary risk factors for thrombophilia in Somalia and identification of a novel Gln544Arg mutation in coagulation factor V

**DOI:** 10.1007/s11239-017-1543-8

**Published:** 2017-09-09

**Authors:** Abshir Ali Abdi, Abdimajid Osman

**Affiliations:** 1 Faculty of Medicine, East Africa University, Bosaso, Puntland Somalia; 2 Division of Clinical Chemistry, Region Östergötland, Ingång 64, 581 85 Linköping, Sweden; 3 Department of Clinical and Experimental Medicine, Linköping University, Linköping, Sweden

**Keywords:** Genotype, Alleles, Venous thrombosis, ABO Blood-Group System, Factor V Leiden, Prothrombin

## Abstract

Thrombophilia, commonly manifested as venous thromboembolism (VTE), is a worldwide concern but little is known on its genetic epidemiology in many parts of the globe particularly in the developing countries. Here we employed TaqMan genotyping and pyrosequencing to evaluate the prevalence of known common nucleotide polymorphisms associated with thrombophilia in a Somali population in the Puntland region of Somalia. We also employed next generation sequencing (NGS) to investigate other genetic variants in a Somali patient with deep venous thrombosis (DVT). As expected, we found no existence of factor V Leiden (rs6025) and prothrombin G20210A (rs1799963) in the Somali population. The G allele of ABO [261G/delG] polymorphism (rs8176719) was found at a frequency of 29%, similar to that observed in other African populations. We found the lowest so far reported frequency of MTHFR C677T (rs1801133) polymorphism in the Somali population (T allele frequency 1.5%). A novel and deleterious single nucleotide variation in exon 11 of coagulation factor V (c.1631A>G) causing Gln544Arg exchange in factor V was identified in a 29 years old Somali female with DVT. The same patient was heterozygous to VKORC1 Asp36Tyr polymorphism (rs61742245) that predisposes to warfarin resistance. In conclusion, this study shows that common hereditary factors for thromboembolism found in Caucasians are either less frequent or absent in the Somali population—similar to the situation in other Africans. NGS is possibly a better choice to detect genetic risk variants for thrombosis in this ethnic group.

## Introduction

Venous thrombosis, usually evolving from thrombophilia and commonly manifested as deep venous thrombosis and pulmonary embolism (VTE), is a major health concern worldwide. There are known strong genetic risk factors for VTE. These involve deficiencies in the innate anticoagulants protein C, protein S and anti-thrombin—typically with low frequencies in the general population due to their severity [1]—but also functional impairments in the pro-coagulants such as fibrinogen, factor V and prothrombin [2]. Blood group non-O is also a known risk factor [3]. The tag single nucleotide polymorphism (SNP) rs8176719 in the ABO locus representing a DEL/G polymorphism [261G/delG] provides either a deletion that generates O blood group and a lower risk for VTE, or alternatively an insertion of a G nucleotide providing A or B antigens and increased risk for VTE [4]. In addition, methylenetetrahydrofolate reductase (MTHFR) C677T polymorphism has been proposed as a plausible candidate for VTE risk [5, 6]. The role of MTHFR C677T (rs1801133; A222V) polymorphism encoding the thermolabile MTHFR in cardiovascular disease is controversial and there is conflicting opinions on its role in thrombosis pathogenesis [7], but it is nevertheless associated with hyperhomocysteinemia, a condition that causes multiple disorders [8]. Further, reports from recent genome-wide association studies (GWAS) indicate that there may be other genetic risk factors for VTE [4, 9].

The two most evaluated genetic risk factors for VTE are factor V Leiden (rs6025; causing protein C resistance) and prothrombin G20210A (rs1799963), each increasing thrombotic risk by threefold to fivefold [2] and synergistically up to 20-fold [10]. The prevalence of these two SNPs is 3–15% in Caucasians but is assumed to be rare in other ethnic populations [2]. There is, however, little genetic data from many parts of Africa—the most genetically diverse continent in the world—necessitating more studies to be performed that explore prevalence of common VTE genetic risk markers in these regions. Particularly in the Horn of Africa where Somalia is located, information on hereditary risk markers for VTE is virtually non-existent.

In the present study a young 29 years old Somali female who was diagnosed for deep venous thrombosis (DVT) in the Östergötland Country of southern Sweden was remitted to the University Hospital in Linköping for genetic analysis. The patient had no factor V Leiden or prothrombin G20210A polymorphism, and by applying next generation sequencing (NGS) we identified a novel coding single nucleotide variation (SNV) in the factor V locus of this patient that was predicted to be critical and might possibly explain her disease. Starting from this point, we sought to evaluate the prevalence of both this novel mutation as well as known common genetic markers for VTE including V Leiden, prothrombin G20210A, ABO 261 [−/G], and methylenetetrahydrofolate reductase (MTHFR) C677T in the Somali population. These SNPs (excepting MTHFR C677T) were found to provide a predictive power for venous thrombosis in Caucasians [11].

## Methods

All genotyping and NGS analysis were carried out at the division of Clinical Chemistry, University Hospital in Linköping, Sweden.

### TaqMan genotyping

Using Catch-All Sample Collection swabs (Epicentre, Madison, WI, USA), buccal cells were collected from unrelated medical students and university staff (n = 101) at the East Africa University in Bosaso city of the Puntland region of northern Somalia. Extraction and purification of DNA was performed with the PureLink Genomic DNA Kits using the standard protocol supplied by the manufacturer (Life Technologies, Carlsbad, CA, USA). TaqMan SNP assays for genotyping factor V Leiden (rs6025), prothrombin G20210A (rs1799963) and MTHFR C677T (rs1801133) were purchased from Life Technologies. TaqMan genotyping was performed in a 25 µl reaction with TaqMan Genotyping Master Mix on a Stepone Plus instrument following assay protocols provided by the manufacturer (Life Technologies).

### Pyrosequencing

A new pyrosequencing [12] method was established for genotyping the O blood group defining [−/G] polymorphism (rs8176719) in exon 6 of the ABO locus using the PCR primers 5-TCGCATTTGCCTCTGGTT-3′ (forward) and B-5′-CGTTGAGGATGTCGATGTTG-3′ (biotinylated reverse primer). The PCR primers amplified a region of 251 base pairs (bp) that was sequenced using the sequencing primer GGAAGGATGTCCTCGT. The sequence-to-analyse was GGT[G]ACCCCTTGGCTGGCTCCCATTGTCT where [G] indicates presence or absence of the variable G-allele. As the ABO locus is rich in tandem repeats, specificity of the PCR primer pairs was verified by UCSC In-Silico PCR (http://genome.ucsc.edu/cgi-bin/hgPcr) and on 2% agarose gel electrophoresis.

A novel c.1631A>G heterozygous mutation in exon 11 (Gln544Arg) of coagulation factor V found in a patient was verified by pyrosequencing using the following PCR and sequencing primers: B-5′-ACTTGGGGTCATCACGTTTC-3′ (forward biotinylated PCR primer), 5′-TCCCTATTGCTTGCTTTTGTCA-3′ (reverse PCR primer), and 5′-GCAGCAGACATCGAA-3′ (sequencing primer). The sequence-to-analyse was CRGCAGGCTGTGTTTGCTGTGTTTGAT where “R” represents the variable position (A/G). All pyrosequencing reactions were run on a PyroMark Q24 instrument using standard reagents and protocols provided by the instrument manufacturer (Qiagen, Hilden, Germany). Allele frequency was calculated by gene counting.

### Next generation sequencing (NGS)

DNA of a thrombosis patient was isolated from EDTA-blood using EZ1 BioRobot (Qiagen). Targeted NGS using HaloPlex Target Enrichment reagents for library preparation (Agilent Technologies, Santa Clara, CA, USA) was applied to sequence coding sequences of 15 loci involved in haemostasis (Table 1). DNA library was sequenced on a miSeq instrument with v2 reagent kit (Illumina Inc., San Diego, CA, USA), following the suppliers’ recommendations. Sequence reads were mapped to the human reference genome (*GRCh37*/hg 19).

**Table 1:** A panel of 15 loci involved in haemostasis that were sequenced by next generation sequencing in DNA from a patient with deep venous thrombosis

Gene name	Approved symbol	Chromosome location
Protein C, inactivator of coagulation factors Va and VIIIa	PROC	2q14.3
Protein S (alpha)	PROS1	3q11.1
Serpin family C member 1 (antithrombin-III)	SERPINC1	1q25.1
Von Willebrand factor	VWF	12p13.31
Coagulation factor II, thrombin	F2	11p11.2
Coagulation factor V	F5	1q24.2
Coagulation factor VII	F7	13q34
Coagulation factor VIII	F8	Xq28
Coagulation factor IX	F9	Xq27.1
Coagulation factor XI	F11	4q35.2
Fibrinogen alpha chain	FGA	4q31.3
Fibrinogen beta chain	FGB	4q31.3
Fibrinogen gamma chain	FGG	4q32.1
Vitamin K epoxide reductase complex subunit 1	VKORC1	16p11.2
Gamma-glutamyl carboxylase	GGCX	2p11.2

### Single nucleotide variation (SNV) repository

A novel SNV, c.1631A>G in coagulation factor V, was submitted to the dbSNP database (https://www.ncbi.nlm.nih.gov/snp/) and is to be publicly available in Build B151 under the sequence identifier number ss2137510516.

## Results

In Table 2, we summarise the genotyping results for common VTE risk factors analysed in the Somali subjects investigated in this study. We found no evidence for existence of factor V Leiden (rs6025) or prothrombin G20210A (rs1799963) variants in Somalia. None of the 101 individuals in Somalia and the thrombosis Somali patient in Sweden carried these SNPs. Indeed, all of the 204 alleles investigated in this study exhibited the ancestral G allele of these two SNPs. This is consistent with the results obtained from other African populations [13] and with that of Abdulkadir et al. [14] who reported absence of factor V Leiden in Ethiopia, a neighbouring country to Somalia [15].

**Table 2:** Information on the SNPs analysed in this study

SNP rs-number (name)	Chromosome position	Sample size (n)	Genotype (frequency %)	Minor allele (frequency %)	Disease/condition associated
rs6025 (Factor V Leiden; G1691A)	chr1:169549811	101	G/G (100) G/A (0) A/A (0)	A (0)	VTE [16–18]
rs1799963 (Prothrombin G20210A)	chr11:46739505	101	G/G (100) G/A (0) A/A (0)	A (0)	VTE [19, 20]
rs1801133 (MTHFR C677T)	chr1:11796321	101	C/C (98) C/T (3) T/T (0)	T (1.5)	VTE [5, 6], foetal neural tube defects and early pregnancy loss [21]
rs8176719 (ABO [261G/delG])	chr9:133257521	101	G/G (15) −/G (28) −/− (58)	G (29)	VTE [22, 23]

**Fig. 1:**
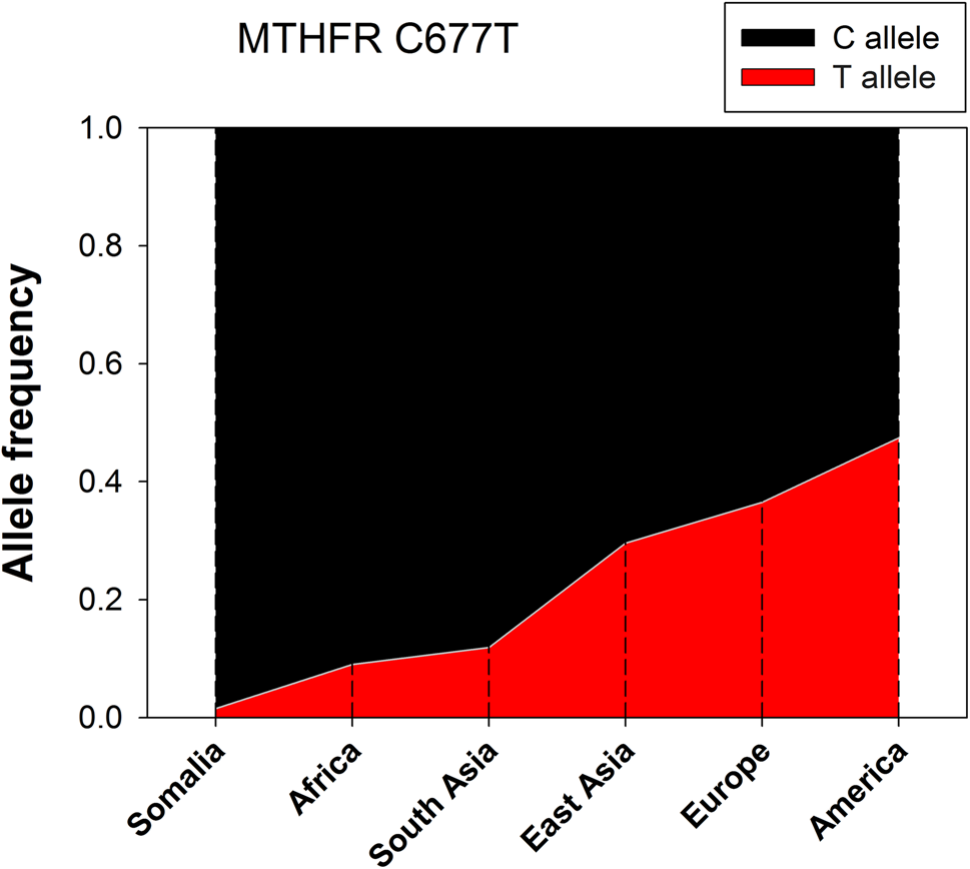
Frequencies of MTHFR C677T alleles in different world populations. The African panel includes other African populations except Somalia. “America” refers to the native Americans. C677 represents the ancestral allele whereas T677 encodes the A222V polymorphism generating the thermolabile MTHFR phenotype

The genotype frequency of MTHFR C677T polymorphism (rs1801133) was low in the Somali population. We identified only three individuals heterozygous to this SNP corresponding to a T allele frequency of 0.015 (1.5%). This indeed represents the lowest T allele frequency of MTHFR C677T SNP found so far in any population when compared with data reported in the 1000 Genomes Project Phase 3 (http://www.internationalgenome.org/home) (Fig. 1) as well as with the MTHFR genotype data obtained with other African populations [24].

**Fig. 2:**
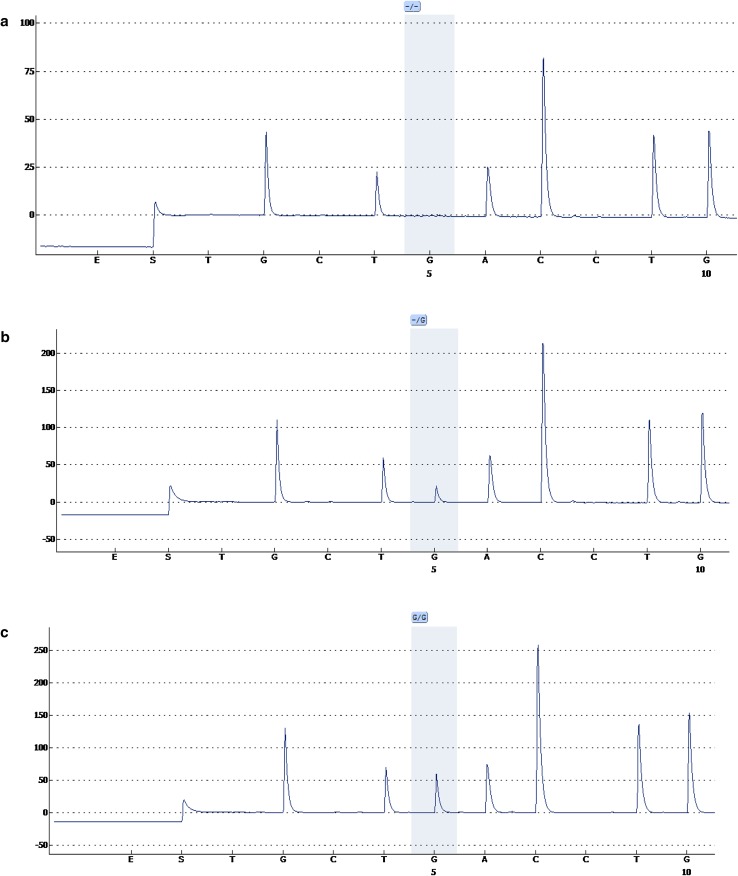
Genotyping of ABO [261G/delG] polymorphism by pyrosequencing. *Shadows* indicate the variable INDEL position (−/G). An individual without the G allele at this position (−/−) is shown on the *upper diagram* (**a**), whereas person in the *middle chart* (**b**) is heterozygous (−/G) and lacks G at this position in one chromosome while the other chromosome has a G insertion. The *bottom diagram* (**c**) shows an individual homozygous to the G allele (G/G)

We used a newly developed pyrosequencing method to genotype the O blood group discriminating [−/G] polymorphism (rs8176719) in exon 6 of the ABO locus. This INDEL polymorphism at codon 261 [261G/delG] is associated with VTE risk [22, 23] and we were therefore interested in its frequency in the Somali population. We found that the G allele frequency of ABO [261G/delG] polymorphism was perfectly similar to that reported in the dbSNP database for other African populations (29%). In addition, frequency of ABO 261 [−/−] genotype representing the O blood group was found to be 58%, which also perfectly matches frequency of the phenotype O blood group found in Somalia three decades ago by Sistonen et al. [25]. Figure 2 illustrates examples of the different ABO [261G/delG] genotypes that were identified in the Somali population.

**Fig. 3:**
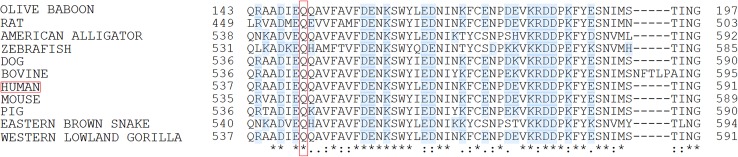
Alignment of a region of the human factor V (residues 537–591) with corresponding proteins of 10 other species. Amino acid position at 544 representing a glutamine (Q544; Gln544) replaced by an arginine (R, Arg) in a thrombosis patient is highlighted with a *red vertical rectangle. Blue shadows* indicate charged residues. *Asterisk* (*) indicates amino acid positions fully conserved across species, whereas a *colon* (:) and a *period* (.) indicate strong and weak conservation, respectively

**Fig. 4:**
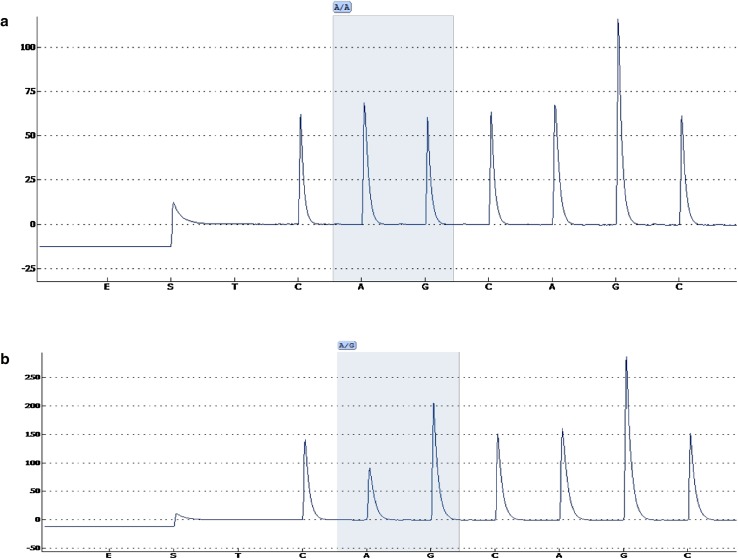
Genotyping of factor V c.1631A>G (Gln544Arg) mutation by pyrosequencing. *Shadows* indicate the variable position (A/G). *Upper diagram* **a** shows homozygosity for the wildtype A allele. The *lower chart* **b** shows a heterozygote variant (A/G) that we identified in a patient with thrombosis

We used NGS to explore genetic variants that might be associated with thrombosis in a patient with DVT. Using a panel comprising 15 loci involved in haemostasis (Table 1), we identified a heterozygous SNV in exon 11 (c.1631A>G) of coagulation factor V in this patient (SNV submitted to the dbSNP database: ss2137510516 in Build B151). The mutation causes substitution of a glutamine residue at position 544 with arginine (Gln544Arg) in the factor V protein. This position is close to the cleavage site of protein C (534–535). Since this variant had not been previously described, we used bioinformatics prediction tools to assess the deleteriousness of this SNV. Two such prediction tools, CADD (Combined Annotation Dependent Depletion) [26] and SIFT (Sorting Intolerant From Tolerant) [27], independently classified the factor V Gln544Arg mutation as deleterious. Further, factor V amino acid alignment revealed that Gln544 is highly conserved across species. Indeed, Gln544 was conserved in all of the 11 species aligned including mammals, reptiles and fish (Fig. 3) suggesting an important function of this amino acid in the factor V protein. We therefore concluded that Gln544Arg variation presumably alter factor V function although its exact role in thrombosis remains to be verified. The c.1631A>G (Gln544Arg) mutation was confirmed by pyrosequencing (Fig. 4). None of the other 101 Somali individuals that participated in this study carried this mutation, implying that it is not frequent in the general population presumably due to its high degree of deleteriousness. In addition to Gln544Arg mutation, we identified VKORC1 Asp36Tyr SNP (rs61742245) in this patient who was heterozygous to this polymorphism. This SNP is common in Ethiopians (allele frequency 15%) as well as in Ashkenazi Jews and is associated with higher (>70 mg/week) warfarin dose requirements [28].

## Discussion

Although VTE is a complex disease, with lifestyle and environmental factors as important elements, the genetic component remains significant. Prevalence of common genetic risk factors for VTE has been investigated in numerous ethnic populations. Vast majorities of these studies were performed in developed western countries, while there is still little data, or no data at all, reported from many developing nations. Somalia is a country that is recovering from more than a quarter century of unrest and refugee crisis. Infectious diseases and emergency medicine consume whatever available of the overstretched and scarce health care resources in the country and thrombosis is not on the public agenda. Since collapse of the central government in 1991 there has been very little medical research conducted in the country and there is no national registry for diseases such as thrombosis. Incidences of thrombosis in Somalia are therefore unknown. Although some research has been performed on Somali refugees living in the west, the genetic panorama of inherited disorders is virtually unexplored in the Somali population. In the current study, we studied the frequency of common genetic markers for thrombosis in the Somali population using a limited number of samples from 101 unrelated volunteers and one thrombosis patient. First, we found that the two most common VTE genetic risk factors in Caucasians, factor V Leiden and prothrombin G20210A, are non-existent, or at least very rare, in the Somali population, supporting the Neolithic farming origin of these SNPs in the Middle East [29]. Ethnic Somalis, like some other ethnic groups in the Horn of Africa, share a significant portion (30–50%) of ancestry with Middle Eastern and North African populations [15, 30] where these two SNPs are common. A recent study found, however, that this admixture occurred before the agricultural revolution [15] which explains why the more recent factor V Leiden and prothrombin G20210A are not found in the Horn of Africa.

The G allele frequency of ABO [261G/delG], a third common VTE risk factor, was found to be similar to that found in other African countries (29%) and lower than in Eurasians. Strikingly, a study performed in 1987 by the Somali Red Crescent Society and the Finnish Red Cross Blood Transfusion Service who analysed 1,026 blood samples of Somalis from the entire country, found that the phenotype O blood group had a frequency of 58% [25], which perfectly matches the genotype frequency (58%) that we found for ABO 261 [−/−] representing the same blood group.

In the Somali population a low T allele frequency of MTHFR C677T polymorphism was observed, indeed lower than in any other world population studied up to now. In addition to its reported role in cardiovascular diseases, MTHFR 677T allele is associated with neural tube defects and pregnancy loss [31]. Remarkably, Mayor-Olea et al. [32] observed an ongoing shift of MTHFR C677T in the Spanish population, where frequency of the T allele has increased during the last 50 years at the expense of C allele. They speculated an existence of a positive genetic selection for the T allele in their population. Whatever benefits or disadvantage the MTHFR 677T allele may have, however, our data shows that this polymorphism is infrequent in the Somali population.

On the whole, our study shows that the most known common genetic risk factors for VTE are either absent or less frequent in the general Somali population when compared with other ethnic populations. However, this study also shows that there may be other genetic risk factors for VTE in the Somali population as we now report for the unique factor V c.1631A>G (Gln544Arg) heterozygote mutation that we found in a young Somali female patient with DVT. Further studies on this mutation will be necessary before its role in VTE can be confirmed. The Gln544 residue is located in a highly conserved region of factor V that is close to the cleavage site of protein C (534–535). Glutamine is a neutral polar amino acid whereas arginine is positively charged and it is therefore not inconceivable that its transition to arginine may affect factor V inactivation and cause protein C resistance.

One limitation of this study is that we genotyped samples collected from one region (Puntland) in Somalia. However, ethnic Somalis were found to be genetically highly homogenous regardless regional or clan affiliation [25, 30] and we have therefore no reason to believe that the genotyping results found in this study are not representative to all Somalis.

Finally, it is our opinion that NGS is the most appropriate option available when investigating genetic bases of VTE in non-Caucasian patients as these individuals usually do not carry the most characterised VTE genetic risk factors (i.e. factor V Leiden and prothrombin G20210A) often included in the standard laboratory analysis for thrombophilia investigation in many countries. With NGS being the leading diagnostic tool for genetic diseases, it is now possible to detect inherited traits associated with both VTE as well as drug response (pharmacogenetic), which enables a more personalised treatment. In the current study, for instance, NGS enabled simultaneous detection of Gln544Arg as well as VKORC1 Asp36Tyr mutations in the same patient. The latter mutation causes warfarin resistance [28], a drug with which the mentioned patient was treated for her DVT.

In conclusion, this study shows that common genetic predictors used to assess risk for VTE in Caucasians are less relevant in the Somali population. Instead we believe that NGS is a more appropriate diagnostic tool for discovery of hereditary thrombosis markers in individuals of non-Caucasian origin.
